# Diachronic Atlas of Comparative Linguistics (DiACL)—A database for ancient language typology

**DOI:** 10.1371/journal.pone.0205313

**Published:** 2018-10-11

**Authors:** Gerd Carling, Filip Larsson, Chundra A. Cathcart, Niklas Johansson, Arthur Holmer, Erich Round, Rob Verhoeven

**Affiliations:** 1 Centre for Languages and Literature, Lund University, Lund, Sweden; 2 Department of Comparative Linguistics, University of Zurich, Zürich, Switzerland; 3 School of Languages and Cultures, University of Queensland, Brisbane St Lucia, Australia; Institute of Computing and Information Technology, PAKISTAN

## Abstract

Feature stability, time and tempo of change, and the role of genealogy versus areality in creating linguistic diversity are important issues in current computational research on linguistic typology. This paper presents a database initiative, DiACL Typology, which aims to provide a resource for addressing these questions with specific of the extended Indo-European language area of Eurasia, the region with the best documented linguistic history. The database is pre-prepared for statistical and phylogenetic analyses and contains both linguistic typological data from languages spanning over four millennia, and linguistic metadata concerning geographic location, time period, and reliability of sources. The typological data has been organized according to a hierarchical model of increasing granularity in order to create datasets that are complete and representative.

## 1. Introduction

The extended Indo-European linguistic area is unique: no other area of the world is richer in documentation of ancient languages. In certain parts, documentation spans over four millennia, something that makes the area important for testing theories on language change and the role of diachrony in explaining linguistic diversity.

The intention of DiACL Typology, a publically available subsection of a database DiACL, a database for comparative and phylogenetic linguistics, also hosting lexical data (https://diacl.ht.lu.se/), is to provide a research data set for the investigation of linguistic diversity with particular utility for the study of diachronic typology. In designing data variables and selecting languages, our aims are as follows:

- to create diachronically informative data sets, suitable for quantitative analysis, characterized by a high degree of granularity within selected linguistic domains, which are known to differentiate linguistic subgroups, and which have few missing data points;
- to select languages within a continuous linguistic area that are phylogenetically representative, both across language families (clades/stocks) and with respect to time-depth, and which are supplemented with metadata including tree topology, time-depth, geographic location, and reliability of the data;
- to focus on linguistic variables that can be assigned meaningful values for most if not all modern languages, and for ancient languages, for which documentary evidence is often limited;
- to organize our feature values according to a model that aims at maximizing the representation of typological variability of individual features.

To meet our desiderata of cross-linguistic applicability and minimal data gaps, we focus on grammatical features pertaining to argument alignment, nominal morphology, tense categories, verbal morphology, and word order. To obtain fine granularity we organize our features in a four-level hierarchy, each level of which expands upon the previous. To ensure that our data set is informative for the study of variation and diachronic stability, we select features that are known from previous research to demonstrate variation at various levels of granularity across the linguistic area selected, and in some cases, whose values are known to exhibit strong correlations between one variable and another.

## 2. Materials and methods

### 2.1. Rationale

The rationale behind DiACL Typology can be summarized as follows: we believe that diachronic typology can be studied independent of reconstructed morphology [1] and that the factors such as genealogy, areal influence, or system-internal pressure can be evaluated statistically based on this data [2–7]. Further, data from precursors of living languages and extinct branches of family trees can give new insights in these questions, if they can be estimated on equal terms with the living languages. We have designed our data sets, our selection of features, and our selection of languages with this specific aim.

Several publically available databases are similar to ours, though they differ slightly in the way they organize features and targeted languages. The most important are WALS—The World Atlas of Language Structures Online (http://wals.info/), SAILS—South American Indigenous Language Structures (http://sails.clld.org/), SSWL—Syntactic Structure of the World’s Languages (http://sswl.railsplayground.net/), and AUTOTYP (http://www.autotyp.uzh.ch/). Another important resource, Grambank (https://www.shh.mpg.de/180672/glottobank), is currently under construction at the Max Planck Institute for the Science of Human History, Jena (https://www.shh.mpg.de/) [8–11]. Our database targets similar domains of typological features as WALS, SAILS, Grambank, AUTOTYP, and SSWL. Differences include, e.g., the level to which data sets are filled, how features are organized hierarchically, whether values are numerical or Boolean, or if features are adapted to language areas or generally valid. None of these databases mentioned before include ancient languages.

DiACL Typology is specific in the following aspects: 1) for typological features, we use a hierarchical model of four levels of increasing granularity of targeted domains of grammar, 2) features are selected to include features that are specific to languages of macro-areas, 3) we include, as far as possible, precursors of living languages as well as extinct branches of language families, 4) we add linguistic metadata (reliability, time frame, geographic location, tree topology, also for extinct and reconstructed languages) that can be used to match linguistic data against extra-linguistic data, retrieved by observation, e.g., for phylogeographical and chronological analyses.

### 2.2. Selection of languages

In DiACL Typology, we have compiled data for the purpose of language diachrony. Data is adapted to areas, and hence, they can be divided into subsets, which embrace a specific language area and allows for including typological properties, which are specific to language areas. In DiACL Typology, the main dataset, Eurasia, targets a continuous language area with a known long history of linguistic records: the Indo-European language continuum (other datasets are small, under construction, and not filled to a satisfactory level). The current paper will deal with the data set Eurasia, which covers 85% of the typological data in DiACL. For extinct languages, there is a correlation between reliability and availability of data: sources of extinct languages are often restricted, sometimes fragmentary, they represent formulaic language (e.g., metrical texts), or texts are translations, in which case typological generalizations can be unreliable [12]. To overcome this problem, we have been forced to constrain our features, so that the modern languages can be quantified on equal terms with the extinct languages. For extinct languages, we use reliable grammatical descriptions and language corpora, for modern languages we use grammatical descriptions as well as language consultants. We have used a combined matrix and questionnaire (S2 Appendix) of hierarchically organized feature values, which we have used for filling in data from grammars as well as for fieldwork.

**Fig 1:**
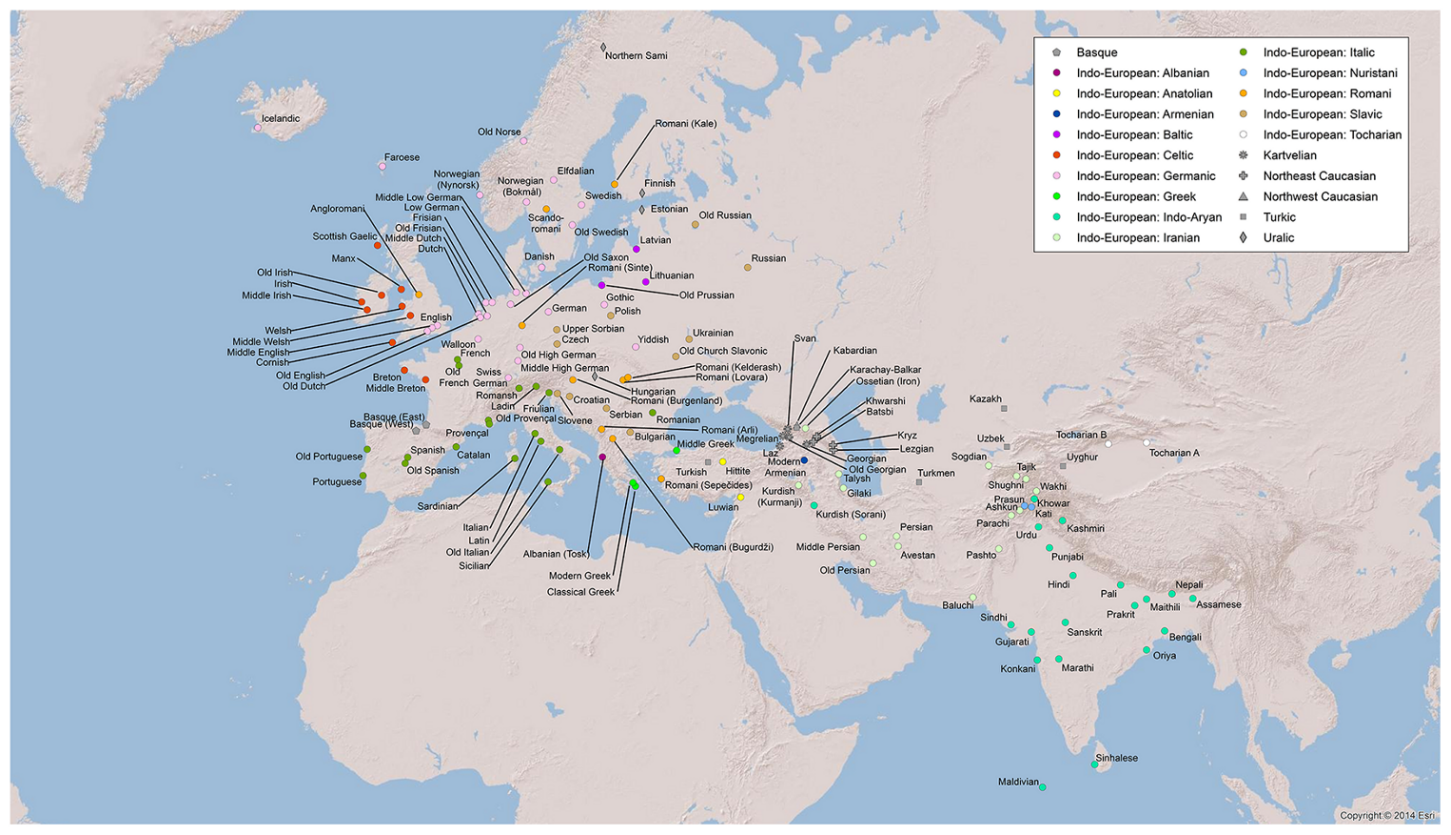
Language map. Location of languages in DiACL Typology/ Eurasia (different colours for different families).

We include Indo-European, adjacent languages from different families, and, as far as possible, earlier states of contemporary languages, dead branches, as well as later stages of migrated languages, from the earliest sources up to the modern period (see Fig 1 and Table 1). For the purpose of testing the impact of areality, we include as many languages as possible from the Indo-Aryan group Romani. These languages have been spoken outside of their original linguistic area, Central India, for 1.5 millennia, and their genealogically closest sister languages within the Indo-Aryan branch are still spoken in Central India [13, 14]. The dialects of Romani are known to have adapted typologically to European languages in various degrees, a procedure that has gone even further in mixed varieties [15].

**Table 1:** Number of languages of each family, type, and time frame in DiACL Typology/ Eurasia (S1 Appendix).

Family	Type	Time frame	Number
**Indo-European**	Archaic	-2000–-500	3
	Ancient	-500–+500	5
	Medieval	+500–+1500	29
	Modern	+1500–+2000	79
	Migratory (Romani)	+1500–+2000	10
**Uralic**	Modern	+1500–+2000	4
**Turkic**	Modern	+1500–+2000	6
**NE Caucasian**	Modern	+1500–+2000	4
**NW Caucasian**	Modern	+1500–+2000	1
**Kartvelian**	Medieval	+500–+1500	1
	Modern	+1500–+2000	4
**Basque**	Modern	+1500–+2000	2
**TOTAL**			148

### 2.3. Principles of selecting and organizing features

Languages, like biological populations, inherit traits with modification from their ancestors, diverge into distinct lineages, go extinct, and engage in horizontal transfer, and accordingly linguistic phylogenetics and allied computational historical methods draw extensively on techniques pioneered in biological systematics [16–22]. However, there may be differences between modern genomic data and the data available to linguists, which can present challenges for quantitative analysis. We mention three challenges and our responses.

Quantitative methods may suffer a loss of power or precision when a data set contains missing values [23, 24]. Therefore, we have tried to maximize the coverage of values by excluding extinct languages with too fragmentary sources, and to adapt the features to match grammars of ancient languages. Accordingly, our data set has a high coverage: the overall coverage is 97.4%, the median coverage for languages is 98% (range 77–100%) and for variables 99% (range 46–100%).

**Fig 2:**
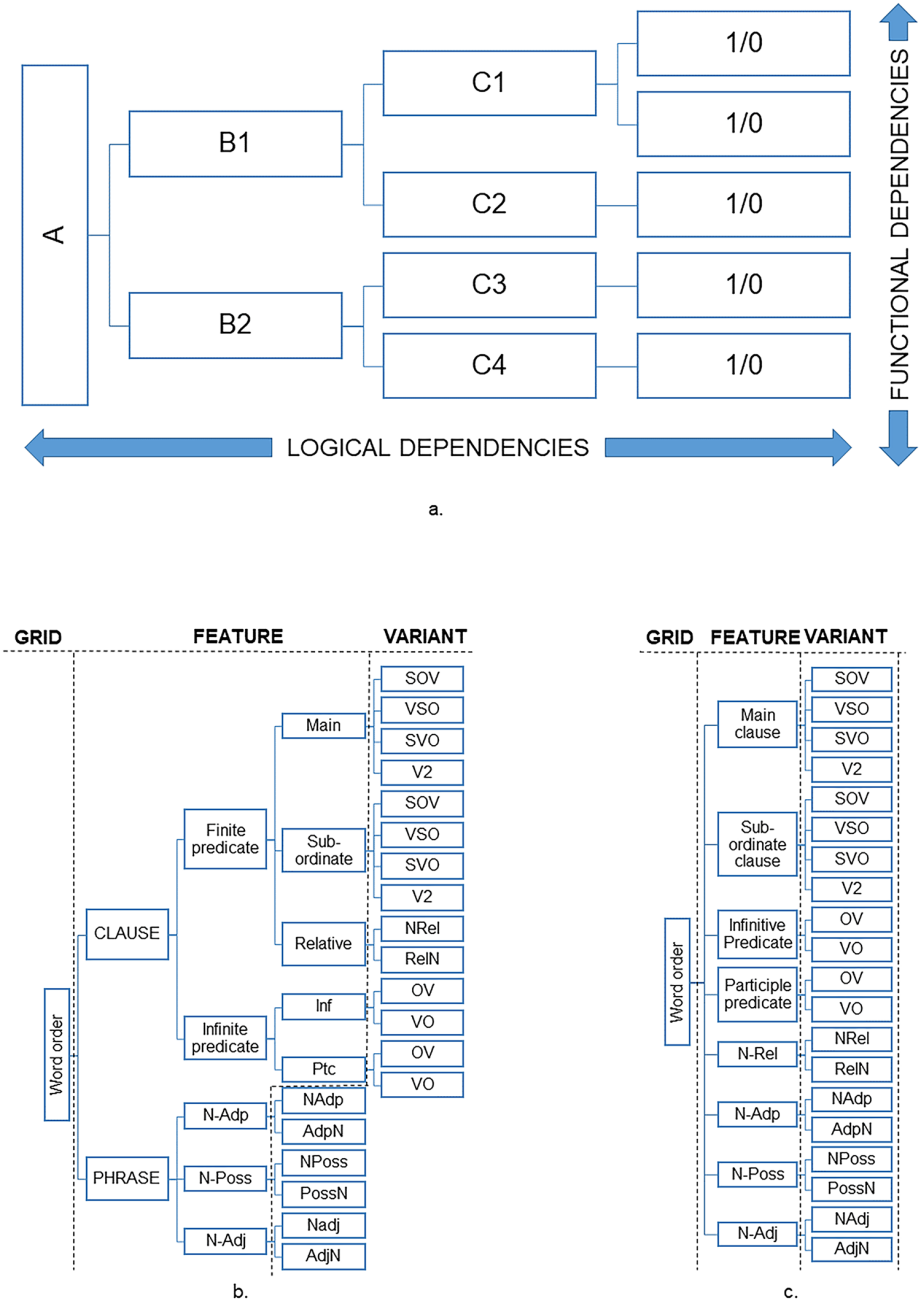
a-c. Organization of typological features. Graphs illustrating the hierarchical principle of organizing linguistic properties, including an prototype model for mapping dependencies in a hierarchical organization of linguistic properties into grids, features, and variants, defined as Boolean values (2a), exemplified on word order classification (2b) merged into a three-level hierarchy (2c).

Unlike genomes, linguistic traits cannot yet be ‘sequenced’; rather, values assigned to linguistic variables are obtained through specialist manual analysis. A repeated observation in the history of linguistics is that such analysis is not deterministic: it may lead to different results from the same observations [25–30]. This is challenging, because a fundamental assumption of automated methods is that a given value of a feature is to be accorded a constant interpretation across the data set. The DiACL Typology/ Eurasia data set addresses this in two ways. First, compilation of the data set by a single coordinating team, in close collaboration with domain experts, has enabled us to exert some control over the commensurability of the codings accorded to the languages. However, a deeper understanding of the language-specific values of codings requires special knowledge: as an aid, we source every data point with a specific reference in literature. Second, our hierarchical-feature approach (see Fig 2) can be understood as an instantiation of the ‘multi-variate’ [31–33] or ‘micro-variate’ [30, 34] approach to language coding, in which one attempts to characterize subtle differences between languages by adapting an increasingly fined-grained approach.

In our data set, features and variants are selected to match the known typological features of the areas included in a targeted macro-area, which in the case of Typology/Eurasia includes Standard Average European (or Charlemagnian) [28, 35, 36], Mediterranean [37], Balkan Sprachbund [38], Circum-Baltic [39], Basque [40], Caucasian [41], and South Asian [42, 43] linguistic areas. Here, we focus on 1) properties that range under the categories alignment, nominal and verbal morphology, and word order, 2) properties that occur within the macro-area and are specific to the linguistic areas described before, 3) properties which ensure a typological variation across the macro-area, 4) properties which tend to correlate typologically in some way, both generally and areally [44], 5) properties that can be identified in a selection of the most well-documented extinct languages.

**Fig 3:**
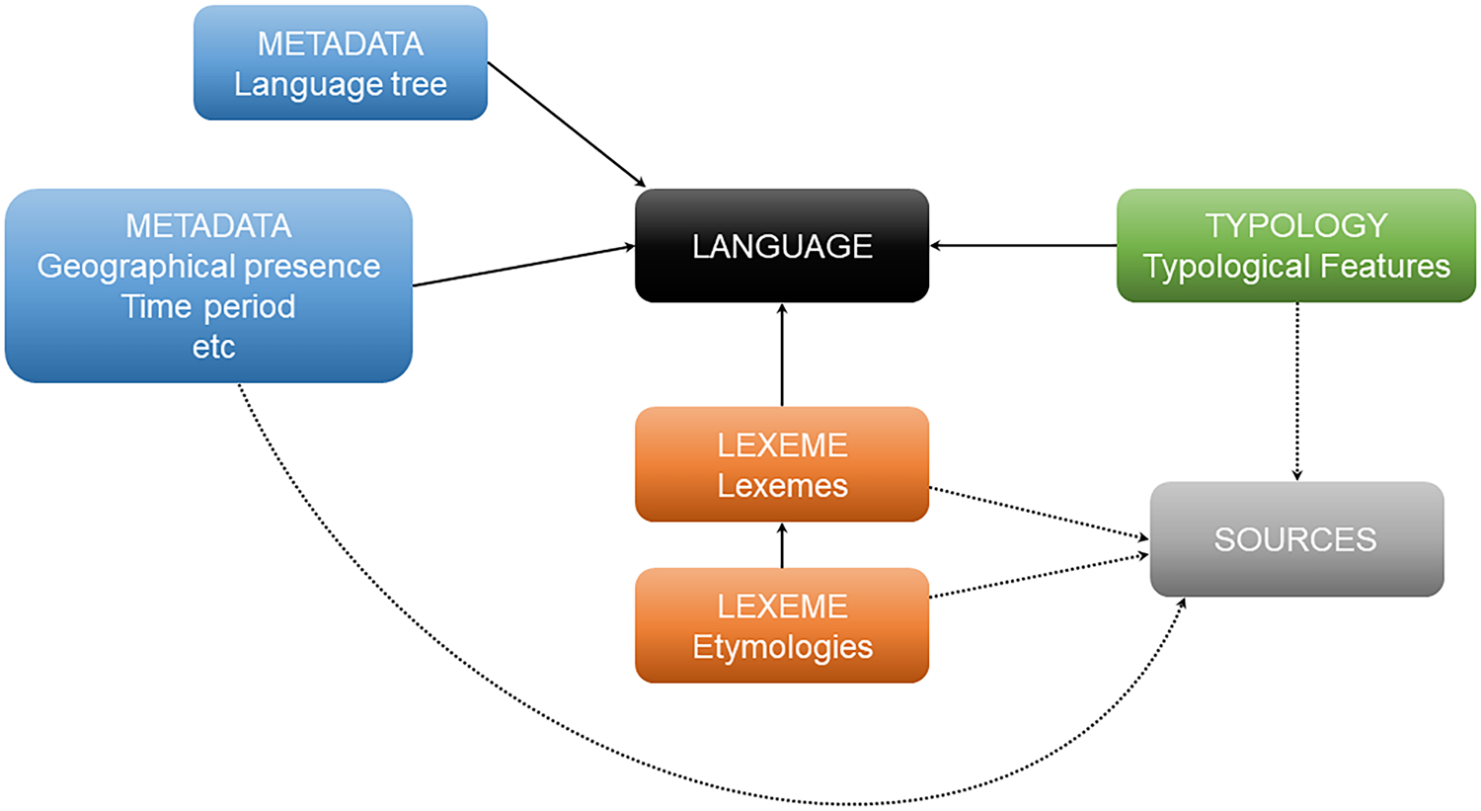
DiACL database overview. Abstract overview of the DiACL database’s general structure.

**Fig 4:**
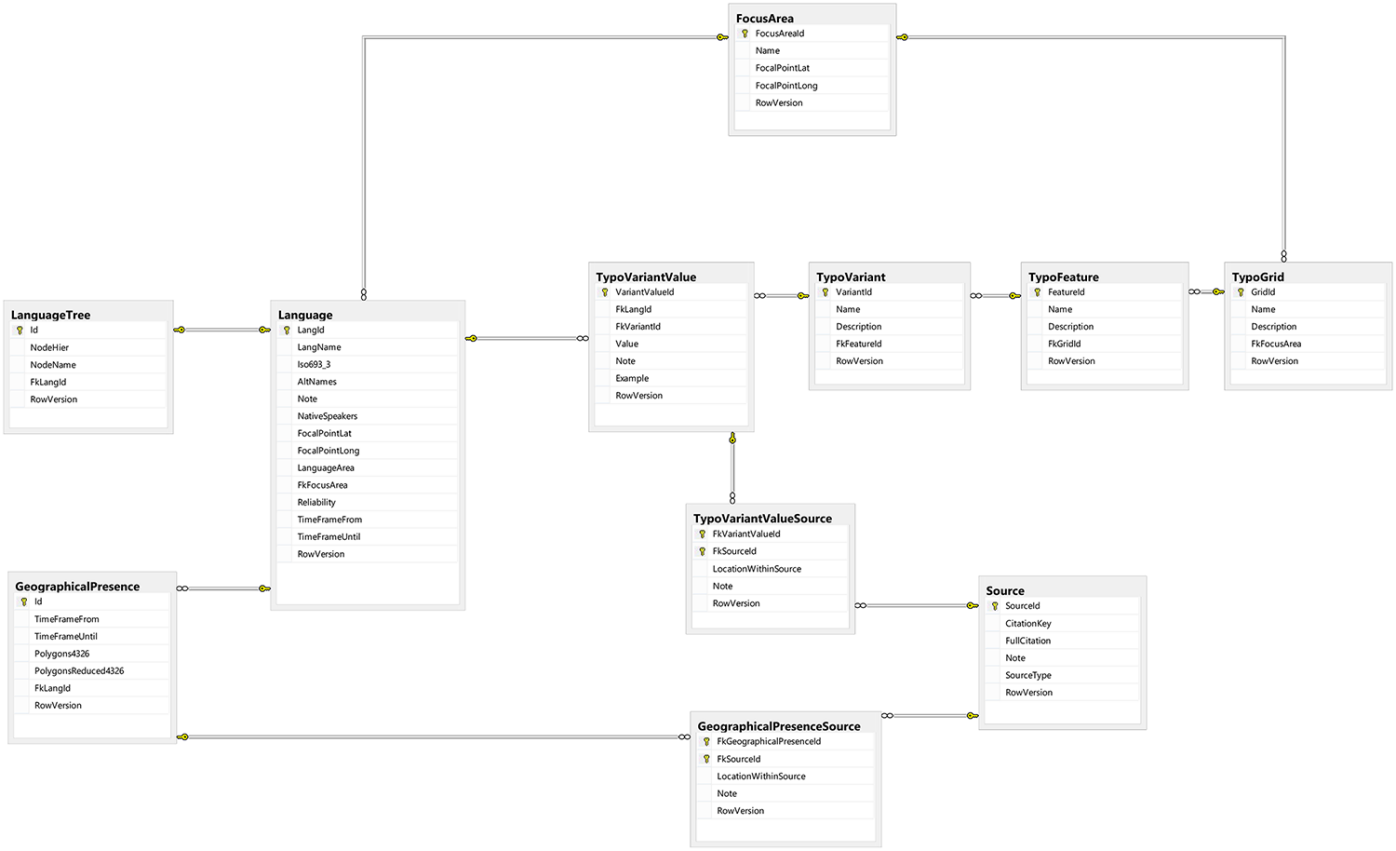
Typology subsection of DiACL. Diagram of the design of the Typology subsection of the database.

Our hierarchical model of grids, features, and variants, which is constrained by the structure of the database (Figs 3 and 4), aims at capturing variation both within the macro-area as well as within individual languages. As an overarching principle, we identify sets of *variants*, by means of a string of values (1/0/NA), the coding of which reflect properties, labelled *features*, of linguistic domains (e.g., alignment, agreement, word order), labelled *grids*. Basically, sets of variants we repeat with respect to other relevant aspects of the targeted grid, such as tense, aspect, morphology, word class, clause type, or typological profile, to construct *features*. In our data, the number of variants of features range from 2–9, and the attested combinations of values (1/0) of features range from 2–47 (see S2 Appendix).

As an example of how features and variants are defined, we can look at word order, exemplified on the situation in German and Irish. It is potentially misleading to classify German as simply verb-second (V2): rather, German is V2 in single-verb main clauses, but OV in infinitival and participial constructions [45]. Irish is not uniformly VSO, but VSO in single-verb main clauses, VO in participial constructions, and OV in infinitival constructions [46]. In our dataset, e.g., word order is divided into 13 features and 31 variants (for details see S2 Appendix). In extinct languages, word order definitions can be complicated. We formulate questions for defining word order (S2 Appendix) as, e.g., “What is the canonical (neutral) word order in a main clause?”. Transferred to an extinct language, statistical results from corpora can be used for a representative coding: if the distribution of, e.g., VO/OV is (hypothetically) 50/50 (or 40/60), the coding is set to 1/1, if the distribution is 30/70 or 20/80, the coding is set to 0/1.

Another example is the coding of alignment (Tables 2 and 3), where the languages in our data set show a great amount of variation. Even though languages such as Basque, Georgian, and Kabardian are normally classified as ergative in a general sense, these languages differ in the way they organize their alignment systems. Basque has an active alignment realized on both nouns and pronouns, which is reflected in the verb morphology but is neutralized for both case and agreement with progressive constructions [47]. Georgian has a tense/aspect split active system, realized on nouns but not on pronouns, combined with an accusative verb agreement alignment [48].

**Table 2:** Explanation of coding variants of alignment [51]. For details see S2 Appendix.

**a)**	A = Sa	The agent (A) of a transitive-active verb bears the same marking as the subject (S) of an intransitive-active verb.
**b)**	Sa = So	The subject (S) of an intransitive-active verb bears the same marking as the subject (S) of an intransitive-stative verb.
**c)**	So = O	The subject (S) of an intransitive-stative verb bears the same marking as the object (O) of a transitive-active verb.

**Table 3:** Samples of coding variants for the alignment feature Noun/ Present progressive in the data, with explanation (N.B.: The list of languages is not complete).

Type	A = Sa	O = So	Sa = So	Example languages	Alignment type
**a)**	1	0	1	Sanskrit, Gothic, Latin, Irish, Icelandic, Tocharian, Lithuanian, Luwian	nominative-accusative
**b)**	1	1	1	Swedish, Danish, French, Kurdish, Breton	no case marking
**c)**	0	0	1	Nepali, Assamese	tripartite
**d)**	0	1	1	Kabardian, Kryz, Khwarshi	ergative
**e)**	1	1	0	Laz, Lezgian	active

For alignment we have, also accounting for the diachronic dimension [49], used a model that aims at describing various aspects of nominative-accusative, active/stative, and ergative marking, starting from the core arguments. For sets of variants we define four correlations, A = O, A = Sa, O = So, and Sa = So [50], for describing the coding relations of A, S, and O with verbs of various transitivity (intransitive, transitive) or semantic (active/stative) types (Sa/So) (Table 2). For distinguishing features we include finer-grained variables which relativize these argument relations within categories of tense/aspect, and morphological realization. For tense/aspect, two distinct categories are selected, past punctual and present progressive. Morphological realization comprises case-marking on full NPs, case-marking on pronouns and agreement pattern on verbs (e.g., are Sa and So marked identically, *by the same morpheme*, *in the same slot*, on the verb?). Accordingly, the result is that an apparently homogeneous categorization such as *active*, *accusative*, *ergative*, or *tripartite* is split up into 8 features and 29 variants, each of which could, in theory, vary independently, although we can identify that they tend to cluster around certain prototypes (Table 3).

Taken together, three feature variants A = Sa, Sa = So, and So = O (Table 2) can describe eight logically possible alignment systems, of which five are attested in our data (Table 3). From this perspective, the variant A = O (S2 Appendix) is redundant.

An important challenge in linguistic research is that typological variables often have mutual dependencies. There is a rich literature on various aspects of this phenomenon, relating to the discussions about the order of meaningful elements across typological properties by Greenberg [52, 53] or typological property correlations by Nichols [5], which is continued into data-driven approaches [54–57] and theories or observations on causality of typological behaviour, synchronically and diachronically [58]. Basically, typological variables may be *logically* dependent on each other, indicating that they target a defined property value or variation, which is dependent on the presence or absence of another property (A is a prerequisite for B, B is dependent on A). In our data, some of these dependencies are implemented in the hierarchical feature model (with the dependent lower in the hierarchy), e.g., the property of WH-initiality (S2 Appendix, 221) is dependent on the language having a WH category, or the property of infinitive word order (S2 Appendix, 231–232) is dependent on the language having an infinitive category. In cases such as these, the coding is indicative: 0/0 implies absence of the category in the language, whereas 1/0, 0/1 or 1/1 codes various types of presence, where the latter coding type is used in case of polymorphic behavior, i.e., that both values of a variant occur in the language. Other dependencies are *functional* [59], such as overt marking across grammatical categories (A and B share grammatical properties). These type of dependencies may be more general, in the sense that they relate to communicative economy, but they may be considerably altered or changed due to diachrony, areality or other random or genealogical factors [56, 59]. In our data, these dependencies are implemented at feature level (Fig 2). An example is the case of alignment systems, which are identified by means of a combination of variants (see below and Tables 2 and 3), and distinguished for tense and word class of the first argument (S2 Appendix, 302–307). Dependency relations in typology can be of several kinds, scaling from stronger to weaker causalities, depending on the nature of the dependency relation [60]. An important part of typological research since Greenberg [53] has dealt with the issue of establishing *implicational* dependencies (if a language has A then it is likely to have B). Basically, these dependencies are empirical, since their identification typically depends on an observation of co-occurrence cross-linguistically, concluded on a larger sample of (often non-genealogical) data. In typological literature, these types of frequencies are often used as an argument of naturalness in human grammar, or ‘universality’ [56].

When compiling a typological data set which organizes typological features hierarchically (where the lowest level is Boolean), an ideal mapping would implement logical features along the hierarchy, to avoid conflicts in the value strings, and to implement functional dependencies crossing over the sub-branches of the hierarchy, in order to enable statistical testing of functional and implicational dependencies (Fig 2a). Even though this mapping is preferred in theory, it is hard to implement in practice, in particular if the numbers of hierarchical levels are given beforehand, as in our case, due to the database structure (Figs 3 and 4). Therefore, we have often been forced to reduce and conflate property dependencies, as in the case of word orders (Fig 2b and 2c).

There are several methods to identify dependencies in the data. For the purpose of computational analysis, dependencies on the level of values, which by downloading come out as a string of independent 1/0/NA values, may be of importance to the user. Dependencies may be both logical, functional, and implicational, but the identification of dependencies should preferably match a postulated research question or hypothesis. As for logical dependencies, we may, at least in theory, identify a number of interdependent variant sets in the data, which either involve attraction (i.e., in order for a variant to be valued 1, one or more additional variants must have that value), or are repellent (i.e., in order for a variant to be valued 1, one or more additional variants must have the value 0. This we may identify for a number of combinations of interdependent variants, such as ‘a language cannot have both full and no A agreement’ (S2 Appendix, 276–277), which can be tested against the data. We tabulate illicit value combinations for these sets of variants (S3 Appendix, both from [61]), and find that for a majority of our postulated sets, illicit combinations are found only in 10% of the character mapping simulations. However, in other of our postulated illicit combinations, the results are not compatible with our assumptions, with 50–60% occurrence of dependencies in the data [61], indicating that (with the exception of case first and case last, S4 Appendix, 12 and S2 Appendix, 260–261), none of our postulated illicit combinations are actually completely absent in our data, and are therefore not logical dependencies in this sense.

The other type is implicational dependency, i.e., the propensity of two variants to co-occur in a language depending on a number of factors, such as economy, universality, language history, or alike. To test this, we quantify two features’ tendency to co-occur using Pointwise Mutual Information (PMI), a measure of association between two events x and y, calculated using the formula PMI(x,y) = log(prob(x,y)/prob(x)prob(y)), i.e., the logarithm of the joint probability of x and y divided by the probabilities of x and y as independent events. If PMI(x,y) > 0, x and y are more likely to occur together than independently [62].

We calculate the PMI for each pair of features in the dataset as follows: prob(x,y) is equal to the number of languages where feature x = 1 and feature y = 1, divided by the number of languages in the sample; prob(x) is equal to the number of languages where feature x = 1, divided by the number of languages in the sample. In the case of missing values, for probabilities of single events like prob(x), we exclude languages where the value of feature x is not known when dividing by the number of languages in the sample (alternatively, the missing value can be changed to the mean of the observed values). For probabilities of co-occurring events like prob(x,y), languages where both feature x and feature y are unknown were excluded, as just described. However, if only one feature value was missing, it is not as clear how to proceed. If feature x = 1 and feature y = ?, we could potentially have feature co-occurrence, if feature x = 0 and feature y = ?, we cannot. A principled approach to dealing with this uncertainty is to exclude languages where feature x = 1 and feature y = ? from the sample, so that they cannot “count against” the overall probability that features x and y co-occur, but retain languages where if feature x = 0 and feature y = ?, since features x and y clearly do not co-occur there, regardless of the missing value. We exclude feature pairs where fewer than 5 languages show feature co-occurrence, given the notorious tendency of low-frequency joint probability events to have inflated PMI values [62]. This leaves us 4547 pairs, organized according to their PMI rank (S4 Appendix).

However, our dataset is mainly diachronic, and the primary goal of the dataset is to enable measuring if typological and morphosyntactic change rates, using a model of ancient data inclusion. Due to the high percentage of Indo-European languages in our data set, this testing of pairwise dependencies yields relatively uninteresting results: most results of high PMI values can be related to the high frequency of specific features, which is an artefact of the high number of SAE languages in our dataset, such as V2 word order or no case marking (A = O). As expected, the results give little information on general or ‘universal’ features.

## 3. Infrastructure

### 3.1. Database DiACL

DiACL Typology is a subsection of a database DiACL (Fig 3), which on the webpage (https://diacl.ht.lu.se/) can be reached via the dropdown menu “Typology” where typological grid for the focus areas, e.g., “Eurasia” can be selected. The general database DiACL contains linguistic data in the form of typological, lexical and etymological data, which represent different sections of the database. Additionally, it contains metadata on languages (see 2.1., 3.2.), common to all sections (typological, lexical, etymological). The central entity of the database is the entity *Language*, which contains languages along with some attributes (see below) and to which all the other sections of the database link. Each *Language* is connected to exactly one particular *FocusArea*, representing a macro-area (i.e., continent) that a language belongs to (*Eurasia*, *Austronesia*, *Amazonia*). The data set targeted in this publication embraces the dataset DiACL Typology/ Eurasia, which represents about 85% of the typological data in DiACL (see 3.3.).

An important additional resource of the database DiACL is constituted by basic vocabulary lists, consisting of a Swadesh 100-list, analysed by cognacy and with loans removed. Nearly all languages for Eurasia that are in the data set DiACL Typology/ Eurasia have complementary sets of basic vocabulary, with the exception of North-East and North-West Caucasian languages, for which cognacy analysis is not available. The basic vocabulary data set has been compiled according to the same basic principles as the typological set: we aim towards symmetry between extinct and contemporary languages (i.e., concerning polymorphism), and all data points are sourced in reliable literature. The basic vocabulary data set is a useful resource, for instance for testing typological against lexical change, or for establishing a lexical phylogenetic tree, against which gain and loss rates of typological data can be measured. The basic vocabulary data can be retrieved from the following URL: https://diacl.ht.lu.se/WordList/Index.

### 3.2. Language and Language metadata

The central entity *Language* of the DiACL database has as its attributes some of the metadata which is stored for each language. The metadata directly stored in the *Language* entity includes the language name (ranging from living languages, such as Swedish, to historical languages, such as Latin, and reconstructed language states, such as Proto-Indo-European). Additionally, alternative language names found in literature may be recorded, and an ISO 693–3 code, if one exists, information on the number of native speakers, as well as the approximate timeframe in which the language was spoken, a categorization of its reliability (distinguished by modern language, dead (fragmentary), dead (well documented), and reconstructed), the general area it belongs to (*Language area*, i.e., Europe, the Middle East, South East Asia, a more fine-grained definition than *Focus area*, see below), and a focal point for pinpointing it on a map. The reliability distinctions of “well-documented” and “fragmentary” approximates the status of extinct languages; they are not a standard of reliability of individual data points. The reliability of any individual data point can be judged by scrutinizing its sources.

Other metadata, such as geographical presence and tree topology is stored in other entities and linked to individual languages by means of unique identifiers. A geographical presence entry for a language comprises a geographical area (in the form of a multipolygon) and a timeframe as its attributes, and is sourced. Such a multipolygon can be downloaded in order to be edited or used in another source. The geographical data points currently in the database have been georeferenced from analogue maps using ArcGIS.

### 3.3. Section Typology

DiACL Typology/ Eurasia is organized under a separate section of the database, *Typology*, the design of which is shown in Fig 4 (all tables related to typology have the prefix *Typo*). In the typology subsection, only attested languages have data (due to the uncertainties connected with typological reconstruction). The tables *TypoGrid*, *TypoFeature*, and *TypoVariant* encapsulate the hierarchy of typological data, described under 2.3. above. As an example, the main category “Nominal morphology” is defined in *TypoGrid*, with a subcategory “Case marking” in *TypoFeature*, with a variant “Case marking obligatory on noun”. Even though Grids refer to universal categories, such as word order, they are constrained to a *FocusArea*, to allow for macro-area specific typological hierarchies. A data point constitutes a value for a variant in a particular language. Such data points are recorded in the table *TypoVariantValue*. Values are Boolean–i.e., to be read as true/false, yes/no, or present/absent. A data point is mandatorily and individually linked to a source, so that no data point can be unsourced. Examples and further notes can also be recorded for a data point. When no data is available in a language for a typological variant, there will simply not be an entry for it in *TypoVariantValue*. In all, the data set DiACL Typology/ Eurasia consists of 17,009 data points, which constitute the majority of the 21,203 data points of the typology section of DiACL (see https://diacl.ht.lu.se/Project/Count).

Through the online interface, editors can improve and check data. Visitors have online access to individual data that can be reached via languages or features (and viewed by their geographic spread). Visitors also have access to an XML encoded extract of all typological data points within a chosen macro area (Link “Download as XML” under “Typological Grid—Index Eurasia”). The structure of the resulting XML file closely follows the data structure of the underlying section of the database (DiACL: Typology: Eurasia), containing the relevant languages, the recorded typological data points within their hierarchical context, and the sources.

### 3.4. Implementation

**Fig 5:**
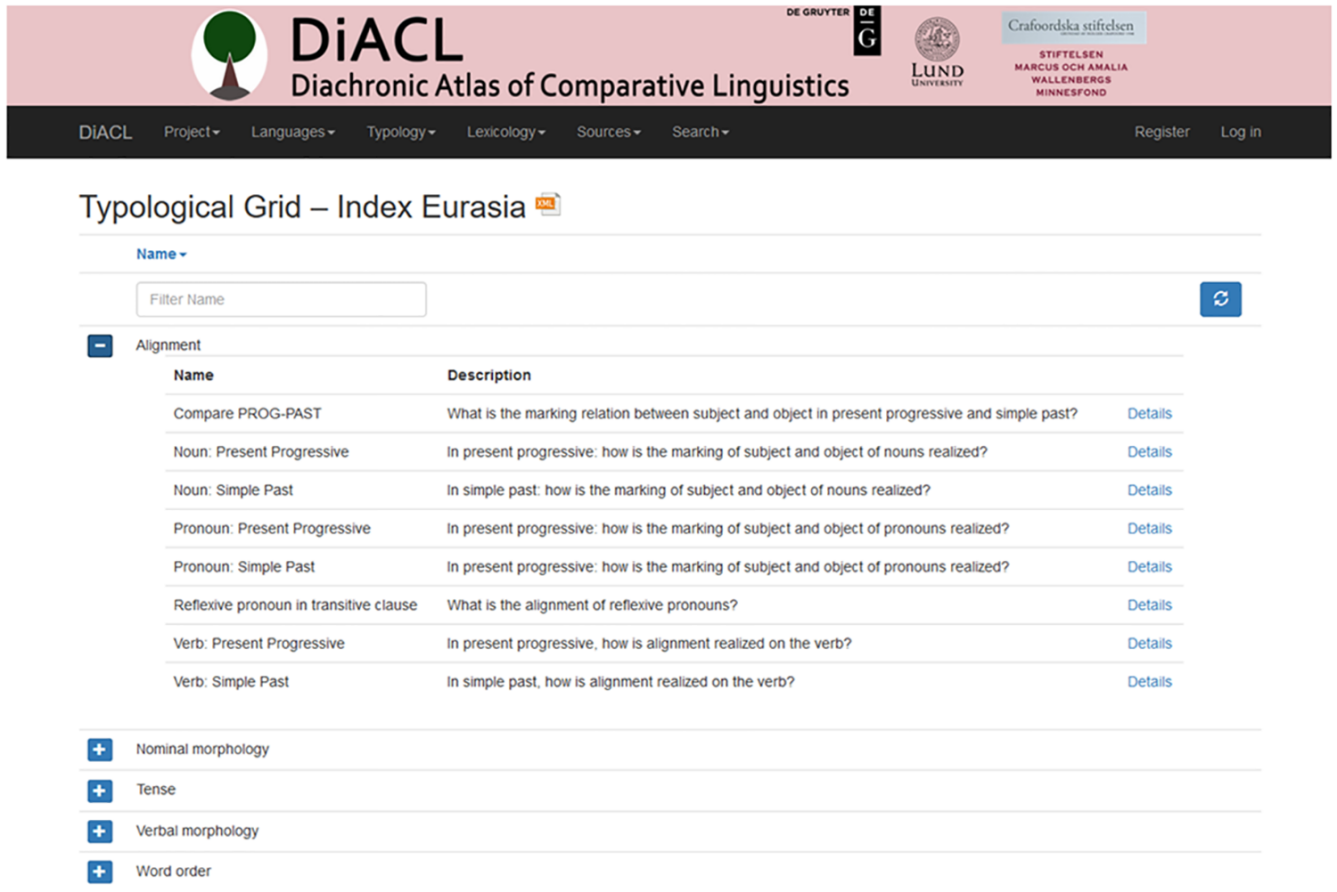
Online interface of DiACL. Screenshot of the interface of DiACL Typology/ Eurasia.

The database resides in Microsoft SQL Server 2014, making use of several of its specialized data types for recording hierarchical and geographical information. Its online interface (Fig 5) has been made in ASP.NET MVC 5 (which on the server side employs the model-view-controller architecture incorporating the *repository* and *unit-of-work* patterns). On the client side, the interface makes use of OpenLayers 3 to display maps and the jQuery library for added responsiveness. Both the database and the online interface reside on an IIS server currently hosted by the Faculty of Humanities and Theology at Lund University. The database is a SWE-CLARIN resource at Lund University (https://sweclarin.se/swe/centrum/lund), located at the Faculty of Humanities and Theology and the Lund Humanities Lab (http://www.humlab.lu.se/en/), a part of CLARIN (http://clarin.eu/), an initiative by ESFRI (http://www.esfri.eu/). The database is also available at SND—Swedish National Data Service (https://snd.gu.se/en/catalogue/study/ext0269). Sustainability of the database is secured through the bodies mentioned before for the coming 10 years. Ongoing discussions aim at integrating the database with the project CLLD—Cross-Linguistic Linked Data (http://clld.org/), hosted by Max Planck Institute for the Science of Human History (http://www.shh.mpg.de/).

## 4. Results

DiACL Typology/ Eurasia can be used for a wide range of quantitative studies. Below, we outline some possibilities for intuitive and simple analyses that can be carried out using the database.

The Boolean data found in DiACL Typology/ Eurasia can be used to calculate pairwise linguistic distance values between languages or features in the data set. The Manhattan distance between each pair of language feature vectors *x* and *y* can be calculated as follows:

$$\sum_{i=1}^{118}\left|x_{i}-y_{i}\right|$$

**Fig 6:**
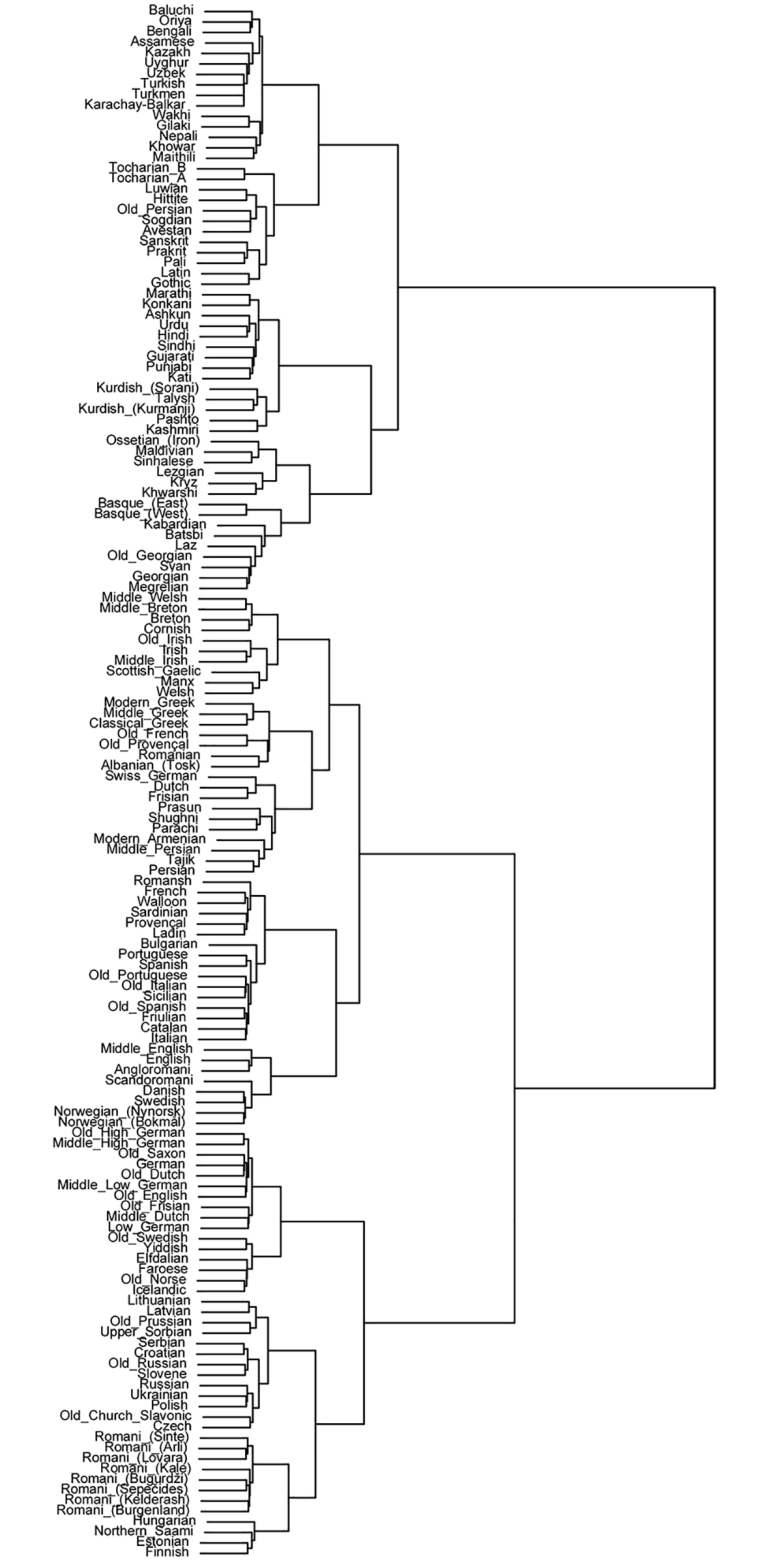
Typological dendrogram of Eurasian languages. Dendrogram of data set DiACL Typology/ Eurasia, based on Manhattan distance values, using hierarchical clustering by means of Ward’s method.

Other popular distance measures such as Euclidean distance can be implemented in programs such as R. A number of techniques can be used to deal with missing data (e.g., the value of a cell with missing data can be set to the mean of all other cells in the vector, or it and its corresponding cell in the second vector can be excluded from computation). There is a great deal of debate regarding the validity of results produced with distance-based methods versus more robust, character-based methods [63, 64]. However, distance-based methods remain a computationally inexpensive way to visualize patterns in linguistic data, and outline hypotheses to test via more computationally intensive methods. Fig 6 shows a dendrogram based on linguistic Manhattan distance values. Hierarchical clustering is carried out using Ward’s method, which seeks to minimize the variance within clusters. Even though this method produces a slightly noisy-looking clustering in some individual cases, a number of interesting patterns emerge.

First, we see a binary division of the languages into a largely Eastern group on one hand, and a largely Western group on the other. Within the Asiatic group, a cluster contains languages of the Caucasus (North-East/ North-West Caucasian and Kartvelian) and Basque (as well as Ossetic and insular Indo-Aryan languages). Another cluster in the Asiatic group contains only archaic and Early Medieval Indo-European languages from Europe and Asia, suggesting an interesting degree of typological affinity between languages belonging to coexistent historical periods (though elsewhere in the dendrogram, precursors typically group with their daughter languages).

Additionally, all Romani dialects are within the European group. While the majority of Romani dialects cluster with Balto-Slavic and Uralic languages, mixed varieties, Angloromani and Scandoromani in our data set, cluster with their respective matrix languages, English and Swedish/Norwegian [15]. There are a number of other interesting patterns in the dendrogram, though some of the more surprising groupings may be artifacts of the distance measure and clustering algorithm used.

**Fig 7:**
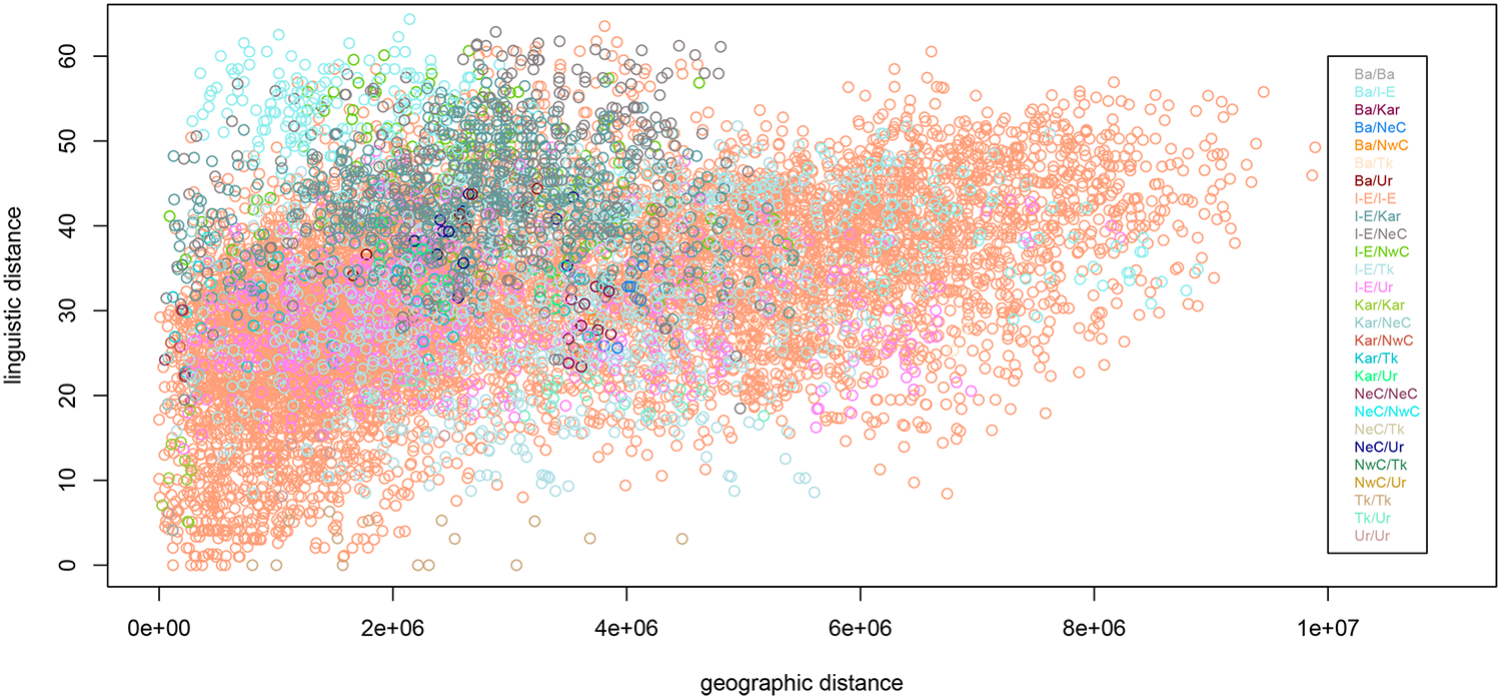
Linguistic distance against geographic distance. Linguistic distance measure plotted against geographic distance (Ba = Basque, I-E = Indo-European, Kar = Kartvelian, NeC = North-East Caucasian, NwC = North-West Caucasian, Tk = Turkic, Ur = Uralic).

Linguistic distance can be modeled as a function of geographic distance, a technique commonly used in dialectometry [65], though studies of this type usually have a narrower scope and concern less disparate language varieties than those included in DiACL Typology/ Eurasia. The DiACL database provides geographic focal points for each language in the sample, and pairwise geographic distances can be calculated from these latitude and longitude values. We use R’s gdistance package [66] to calculate the great-circle (alternatively, as-the-crow-flies) distance between each pair of languages. It is additionally possible to calculate more sophisticated geospatial distance measures (e.g., least-cost distance), but research shows that the effect of terrain-based cost distances on linguistic variation tends to be detectable only at small geographic scales [67, 68]. Fig 7 shows our linguistic distance measure plotted as a function of great-circle distance. Point colors represent differentials between language families (i.e., Indo-European vs. Uralic). It is clear that the picture is quite noisy, and that a number of factors other than geography must explain a great deal of the variance. Nevertheless, the overall picture shows that there is a clear association between the two variables.

**Fig 8:**
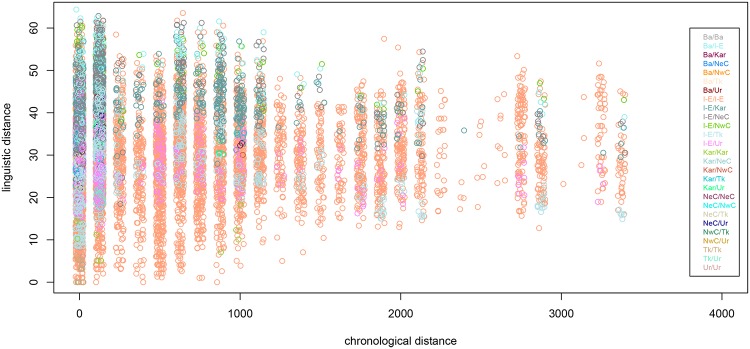
Linguistic distance against chronological distance. Linguistic distance plotted against chronological distance (Ba = Basque, I-E = Indo-European, Kar = Kartvelian, NeC = North-East Caucasian, NwC = North-West Caucasian, Tk = Turkic, Ur = Uralic).

Additionally, we wish to consider the effect of chronological distance on linguistic distance. While the cyclic nature of language change means that languages do not become infinitely dissimilar as chronological distance between them increases, it is nevertheless the case that attested Eurasian ancient and medieval languages are highly dissimilar from most modern speech varieties in terms of typology, and this dissimilarity is visualizable. Fig 8 shows linguistic distance plotted as a function of chronological distance, which is the difference between the mean dates of attestation of two languages (Manhattan and Euclidean distance measures give the same result). The plot shows a weak trend in which linguistic distance increases as chronological distance increases; however, the association is highly heteroscedastic, with higher variance in linguistic distance for lower values of chronological distance. This is undoubtedly an artifact of our sample: the majority of languages are modern languages, and exhibit high typological diversity, whereas ancient and medieval languages are not as well attested and exhibit lower typological diversity. It is therefore not surprising that two contemporary languages in the sample could be more dissimilar than two languages with higher chronological distance between them.

**Fig 9:**
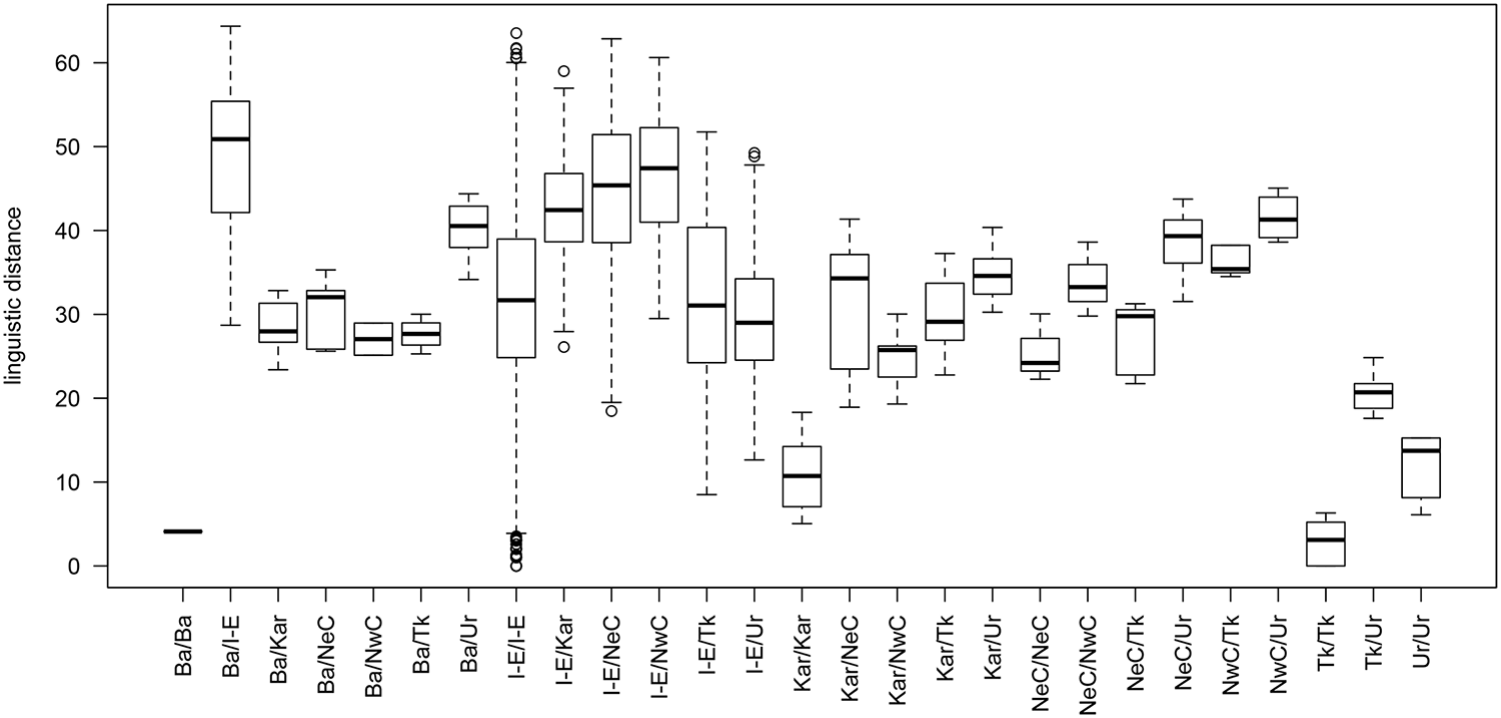
Linguistic distance of language families. Boxplot showing linguistic distance values as a function of language family differentials (Ba = Basque, I-E = Indo-European, Kar = Kartvelian, NeC = North-East Caucasian, NwC = North-West Caucasian, Tk = Turkic, Ur = Uralic).

Using what information we have, we can construct a linear model which seeks to explain the linguistic variation documented in DiACL Typology/ Eurasia. We treat linguistic distance as our response variable. We employ geographic and chronological distance measures as predictors, as well as other variables. For maximum explanatory value, we include language family differential as a categorical predictor. A boxplot showing linguistic distance values as a function of language family differential is seen in Fig 9. We also include an interaction term between geographic and chronological distance, since for a particular level of chronological distance between languages, geographic distance may have a stronger or weaker effect on linguistic distance.

Geographic distance, chronological distance, and the interaction term all have a highly significant effect (*p* < .001). The interaction term, while significant, is quite close to zero, and is also negative (-7.848e-12), indicating that as temporal disparity between languages increases, a weaker effect of geographic distance on linguistic distance is seen (or vice versa). Most distance-based studies observe (and understandably so) that at a certain point, linguistic distance levels off, even as geographic distance increases [65, 69]. With respect to typological features, languages can exhibit dissimilarity only up to a point, i.e., mismatch for, roughly speaking, no greater than 65 out of our 118 features.

Most of the language family differentials are significant as well, with the exception of the following: Basque to Kartvelian, Northeast Caucasian, Northwest Caucasian, and Turkic; Kartvelian to Kartvelian; Turkic to Turkic and Uralic; and Uralic to Uralic. 36% of the variance is explained; roughly 20% of which is contributed by the language family differential predictors.

These analyses provide a small taste of the wide range of investigations that can be carried out using the DiACL Typology/ Eurasia data set. Distance measures can additionally be computed on the basis of individual grids, rather than all typological features, to see how systems vary with respect to each other.

## 5. Conclusion

The database DiACL and the dataset Typology/ Eurasia offer a unique possibility for testing a wide range of parameters as influencing language typology. Most importantly, the high level of granularity and the representative selection of typological features, in combination with a systematic inclusion of data from ancient languages, are valuable resources for testing hypotheses on language evolution and change. In particular, the inclusion of Medieval and Romani languages, besides ancient and archaic languages, serve as crucial intermediate levels for observing changes over longer periods. The rendering of typological features as strings of values (1/0), each representing a fine-grained generalization of typological structure, is highly valid for testing language evolution and change at a very detailed level. In addition, basic vocabulary data of languages included in the typological data serve as a complementary resource, which can be used for a contrast.

In the current paper, which mainly aims to describe the database and the dataset, we can observe a number of new insights emerging from analyses of our data. As for internal dependencies between features, an area of particular interest to cross-linguistic typology, we conclude that dependencies in our data (measured by Pointwise Mutual Information) are highly governed by our areal restriction to Eurasia, as well as our dominance of Indo-European languages. The most frequently co-occurring features are features that dominate Eurasian typological areas. We notice that typological features cluster (in a dendrogram based on Manhattan distance values, Ward’s method) according to geographical distribution rather than phylogeny. In addition, results suggest an interesting degree of typological affinity between languages of coexistent historical periods. As for linguistic distance in correlation to geographic and chronological distance (measured by R’s ggdistance package and Manhattan and Euclidean distance measures), we notice some trends: the picture is noisy, with potential competing explanations for linguistic similarity or distance, but the overall correlation between linguistic and geographic/chronological distance is highly significant (*p <* .001). This implies that in general, linguistic distance increases with increasing distance in space and time. As a result of this process, but also as a result of our available sources (extinct languages are mainly Indo-European), contemporary languages in our data are by far more divergent and variating than any of the historical language states. This is particular the case with the Indo-European family, which is the most geographically and chronologically extended family in our data. History, geography, genetic pressure and numerous additional factors form a complex and dynamic web, which influences linguistic structure; this relationship is worthy of investigation. For the future, we look forward to further quantitative studies that can be carried out using the DiACL database.

## Supporting information

S1 Appendix List of languages and language metadata. (XLSX)

S2 Appendix List of typological grids, and feature variants with description and ID number. (XLSX)

S3 Appendix Blocks of interdependent feature variants. (TXT)

S4 Appendix List of PMI ranks of feature variant pairs. (XLSX)

## References

[1] Viti C. Historical syntax: Problems, materials, methods, hypotheses In: Viti C, editor. Perspectives on Historical Syntax. Amsterdam—Philadelphia: John Benjamins; 2015 p. 3–34.

[2] Jaeger TF, Graff P, Croft W, Pontillo D. Mixed effect models for genetic and areal dependencies in linguistic typology. Linguistic Typology. 2011;15(2):281–319. 10.1515/LITY.2011.021 .

[3] Daumé H, editor Non-parametric Bayesian Areal Linguistics. Human Language Technologies: The 2009 Annual Conference of the North American Chapter of the ACL; 2009; Boulder, Colorado: Association for Computational Linguistics.

[4] Dediu D, Levinson SC. Abstract Profiles of Structural Stability Point to Universal Tendencies, Family-Specific Factors, and Ancient Connections between Languages. PLoS ONE. 2012;7(9):e45198 10.1371/journal.pone.0045198 23028843 PMC3447929

[5] Nichols J. Linguistic diversity in space and time: Linguistic diversity in space and time. Chicago: Univ. of Chicago Press; 1992.

[6] Levinson SC, Greenhill SJ, Gray RD, Dunn M. Universal typological dependencies should be detectable in the history of language families. Linguistic Typology. 2011;15(2):509–34. 10.1515/LITY.2011.034 .

[7] Dunn M, Greenhill SJ, Levinson SC, Gray RD. Evolved structure of language shows lineage-specific trends in word-order universals. Nature. 2011;473(7345):79–82. http://www.nature.com/nature/journal/v473/n7345/abs/10.1038-nature09923-unlocked.html#supplementary-information. 10.1038/nature09923 21490599

[8] The World Atlas of Language Structures Online [Internet]. Max Planck Institute for Evolutionary Anthropology. 2013.

[9] Gray R. Think Big!—The bright future of linguistics. Diversity Linguistics: Retrospect and Prospect; MPI EVA’s Department of Linguistics, May 1–3, 20152015.

[10] Syntactic Structures of the World’s Languages [Internet]. 2011. http://sswl.railsplayground.net/.

[11] Skirgård H, Meer Svd. The Nijmegen Typological Survey (NTS). Language Comparison with Linguistic Databases: RefLex and Typological Databases; Nijmegen, The Netherlands2014.

[12] Haug D. Treebanks in historical linguistic research In: Viti C, editor. Perspectives on Historical Syntax. Amsterdam: John Benjamins; 2015 p. 187–202.

[13] Matras Y. Romani: a linguistic introduction: Cambridge: Cambridge University Press, 2002.

[14] Matras Y. 13 Romani. The Languages and Linguistics of Europe A Comprehensive Guide2011.

[15] Carling G, Lindell L, Ambrazaitis G. Scandoromani: remnants of a mixed language: Leiden: Brill, 2014.

[16] Gray RD, Jordan FM. Language trees support the express-train sequence of Austronesian expansion. Nature. 2000;405(6790):1052–5. 10.1038/35016575 10890445

[17] Pagel M. The history, rate and pattern of world linguistic evolution In: Knight C, Studdert-Kennedy M, Hurford J, editors. The evolutionary emergence of language: social function and the origins of linguistic form. Cambridge: Cambridge University Press; 2000 p. 391–416.

[18] Gray RD, Atkinson QD. Language-tree divergence times support the Anatolian theory of Indo-European origin. Nature. 2003;426(6965):435–9. http://www.nature.com/nature/journal/v426/n6965/suppinfo/nature02029_S1.html. 10.1038/nature02029 14647380

[19] Luay N, Ringe D, Warnow T. Perfect Phylogenetic Networks: A New Methodology for Reconstructing the Evolutionary History of Natural Languages. Language. 2005;81(2):382–420.

[20] Bouckaert R, Lemey P, Dunn M, Greenhill SJ, Alekseyenko AV, Drummond AJ, et al Mapping the Origins and Expansion of the Indo-European Language Family. Science. 2012;337(6097):957–60. 10.1126/science.1219669 22923579 PMC4112997

[21] Bowern C, Atkinson Q. Computational phylogenetics and the internal structure of Pama-Nyungan. Language. 2012;88(4):817–45.

[22] Chang W, Cathcart C, Hall D, Garrett A. Ancestry-constrained phylogenetic analysis supports the Indo-European steppe hypothesis. Language. 2015;91(1):194–244.

[23] Huelsenbeck JP. When are Fossils Better than Extant Taxa in Phylogenetic Analysis? Systematic Biology. 1991;40(4):458–69. 10.1093/sysbio/40.4.458

[24] Wiens JJ, Morrill MC. Missing Data in Phylogenetic Analysis: Reconciling Results from Simulations and Empirical Data. Systematic Biology. 2011 10.1093/sysbio/syr025 21447483

[25] Chao YR. The non-uniqueness of phonemic solutions of phonetic systems. Bulletin of the Institute of History and Philology, Academica Sinica 1939;4(4):36–397.

[26] Lass R. Vowel system universals and typology: prologue to theory. Phonology. 1984;1:75–111.

[27] Dresher BE. The contrastive hierarchy in phonology. Cambridge;: Cambridge University Press; 2009.

[28] Haspelmath M. Comparative concepts and descriptive categories in crosslinguistic studies. Language. 2010;86(3):663–87.

[29] Hyman L. What (else) depends on phonology?. Dependencies Among Systems of Language workshop; Château de la Poste, Ardennes2014.

[30] Round ER, Macklin-Cordes JL. On the design, in practice, of typological microvariables. New Developments in the Quantitative Study of Languages; Helsinki2015.

[31] Bickel B, Nichols J. Autotypologizing Databases and their Use in Fieldwork. In: Austin P, Dry H, Witternburg P, editors. Proceedings of the International LREC Workshop on Resources and Tools in Field Linguistics, Las Palmas. Nijmegen: ISLE and DOBES; 2002.

[32] Bickel B. Typology in the 21st century: Major current developments. Linguistic Typology. 2007;11(1):239–51. 10.1515/LINGTY.2007.018

[33] Bickel B. Capturing particulars and universals in clause linkage. In: Bril I, editor. Clause Linking and Clause Hierarchy: Syntax and Pragmantics. Amsterdam: Benjamins; 2010. p. 51–101.

[34] Round ER. Big data typology and linguistic phylogenetics: design principles for valid datasets. 21st Manchester Phonology Meeting; Manchester2013.

[35] Dahl Ö, Koptjevskaja-Tamm M. The Circum-Baltic languages: typology and contact: Amsterdam; Philadelphia: John Benjamins Pub. Co, 2001.

[36] Auwera Johan van d. 15 Standard Average European. The Languages and Linguistics of Europe A Comprehensive Guide. Berlin—Boston: Mouton de Gruyter; 2011. p. 291–306.

[37] Sansò A. 18 Mediterranean languages. The Languages and Linguistics of Europe A Comprehensive Guide2011.

[38] Mišeska Tomić O. 16 Balkan Sprachbund features. The Languages and Linguistics of Europe A Comprehensive Guide2011.

[39] Wälchli B. 17 The Circum-Baltic languages. The Languages and Linguistics of Europe A Comprehensive Guide2011.

[40] Haase M. 10 Basque. The Languages and Linguistics of Europe A Comprehensive Guide2011.

[41] Daniel M, Lander Y. 6 The Caucasian languages. The Languages and Linguistics of Europe A Comprehensive Guide2011.

[42] Ebert KH. Südasien als Sprachbund In: Haspelmath M, editor. Language Typology and Language Universals: an International Handbook. Handbücher zur Sprach- und Kommunikationswissenschaft. 1 Berlin: De Gruyter; 2001 p. 1529–39.

[43] Masica CP. The Indo-Aryan languages. Cambridge: Cambridge University Press; 1991.

[44] Dryer MS. Greenbergian Word Order Correlations. Language. 1992;68(1):81–138.

[45] Engel U. Deutsche Grammatik. Heidelberg: Julius Groos; 1988.

[46] Ó Siadhail M. Modern Irish grammatical structure and dialectal variation. Cambridge: Cambridge Univ. Press; 1989.

[47] Laka I. Deriving split ergativity in the progressive In: Johns A, Massam D, Ndayiragije J, editors. Ergativity Emerging Issues. Dordrecht: Springer; 2006 p. 173–95.

[48] Vamling K. Complementation in Georgian. Lund: Lund Univ. Press; 1989.

[49] Bauer B. Archaic syntax in Indo-European: the spread of transitivity in Latin and French. Berlin: Mouton de Gruyter; 2000.

[50] Dixon RMW. Basic linguistic theory Vol. 2, Grammatical topics. Oxford: Oxford University Press; 2010.

[51] Dixon RMW. Basic linguistic theory Vol. 1, Methodology. Oxford: Oxford University Press; 2010.

[52] Greenberg JH. Universals of language report of a conference held at Dobbs Ferry, New York, April 13–15, 1961. Cambridge, Mass.: MIT Press; 1963.

[53] Greenberg JH. Language universals: with special reference to feature hierarchies: The Hague: Mouton, 1966.

[54] Bickel B. Capturing particulars and universals in clause linkage: a multivariate analysis In: Bril I, editor. Clause Linking and Clause Hierarchy: Syntax and Pragmatics. Amsterdam—Philadelphia: John Benjamins; 2010 p. 51–101.

[55] Cysouw M. Understanding transition probabilities. Linguistic Typology. 2011;15(2):415–31. 10.1515/LITY.2011.028 .

[56] Hammarström H, O’Connor L. Dependency-sensitive typological distance In: Borin L, Saxena A, editors. Appraches to Measuring Linguistic Differences. Berlin—Boston: Mouton de Gruyter; 2013 p. 329–52.

[57] Blási DE, Roberts SG. Beyond binary dependencies in language structure In: Enfield N, editor. Dependencies in Language. 117–128 Berlin: Language Science Press; 2017.

[58] Dediu D, Cysouw M. Some Structural Aspects of Language Are More Stable than Others: A Comparison of Seven Methods. PLOS ONE. 2013;8(1):e55009 10.1371/journal.pone.0055009 23383035 PMC3557264

[59] Cristofaro S. Implicational universals and dependencies In: Enfield NJ, editor. Dependencies in language. Berlin: Language Science Press; 2017 p. 9–23.

[60] Enfield NJ. Dependencies in language On the causal ontology of linguistic systems. Berlin: Language Science Press; 2017.

[61] Cathcart C, Carling G, Larsson F, Johansson N, Round ER. Areal pressure in grammatical evolution. Diachronica. 2018;35(1):1–34.

[62] Manning CD, Schütze H. Foundations of statistical natural language processing. Cambridge, Mass.: MIT Press; 1999.

[63] Nichols J, Warnow T. Tutorial on computational linguistic phylogeny. Language and Linguistics Compass. 2008;2:760–820.

[64] Dunn M. Language phylogenies In: Bowern CaBE, editor. The Routlegde Handbook of Historical Linguistics. Florence: Routledge; 2014 p. 190–211.

[65] Séguy J. La relation entre la distance spatiale et la distance lexicale. Revue de Linguistique Romane. 1971;35:335–57.

[66] van Etten J. R package gdistance: distances and routes on geographic grids. 2012.

[67] Gooskens C, editor Norwegian dialect distances geographically explained. Language Variation in Europe Papers from the Second International Conference on Language Variation in Europe ICLAVE; 2004; Uppsala: Department of Scandinavian Languages, Uppsala University.

[68] Haynie H. Studies in the History and Geography of California Languages: University of California, Berkeley; 2012.

[69] Nerbonne J. Data-driven dialectology. Language and Linguistics Compass. 2009;3:175–98.

